# Decoding the Immune Microenvironment of Clear Cell Renal Cell Carcinoma by Single-Cell Profiling to Aid Immunotherapy

**DOI:** 10.3389/fimmu.2022.791158

**Published:** 2022-06-24

**Authors:** Jie Liu, Jiangfan Xu, Tong Zhang, Kailong Xu, Peihua Bao, Zhibo Zhang, Kaiwen Xue, Ruyi He, Lixin Ma, Yang Wang

**Affiliations:** ^1^ State Key Laboratory of Biocatalysis and Enzyme Engineering, School of Life Sciences, Hubei University, Wuhan, China; ^2^ College of Intelligent Systems Science and Engineering, Harbin Engineering University, Harbin, China; ^3^ Department of Cardiothoracic Surgery, The 78th Group Army Hospital of Chinese People's Liberation Army, Mudanjiang, China; ^4^ College of Chemistry and Chemical Engineering, Hubei University, Wuhan, China; ^5^ School of Life Science and Technology, Wuhan Polytechnic University, Wuhan, China

**Keywords:** single cell, tumor microenvironment, biomarker, immunotherapy, clear cell renal cell carcinoma

## Abstract

Clear cell renal cell carcinoma (ccRCC) is the most common subtype of kidney cancer, and it is the major cause of kidney cancer death. Understanding tumor immune microenvironments (TMEs) is critical in cancer immunotherapies. Here, we studied the immune characterization at single-cell resolution by integrating public data of ccRCC across different tissue types, and comparing the transcriptome features and tumor TME differences in tumors, normal adjacent tissue, and peripheral blood. A total of 16 different types of cell components of ccRCC were identified. We revealed that there is an overall increase in T-cell and myeloid populations in tumor-infiltrated immune cells compared to normal renal tissue, and the B-cell population in the tumor showed a sharp decrease, which indicates that the cells in tumor tissue undergo strong immune stress. In addition, the cell–cell communication analysis revealed specific or conserved signals in different tissue types, which may aid to uncover the distinct immune response. By combining and analyzing publicly available ccRCC bulk RNA-seq datasets, 10 genes were identified as marker genes in specific cell types, which were significantly associated with poor prognosis. Of note, UBE2C, which may be a good indicator of tumor proliferation, is positively associated with reductions in overall survival and highly associated with tumor grade. Our integrated analysis provides single-cell transcriptomic profiling of ccRCC and their TME, and it unmasked new correlations between gene expression, survival outcomes, and immune cell-type components, enabling us to dissect the dynamic variables in the tumor development process. This resource provides deeper insight into the transcriptome features and immune response of ccRCC and will be helpful in kidney cancer immunotherapy.

**Graphical Abstract:**
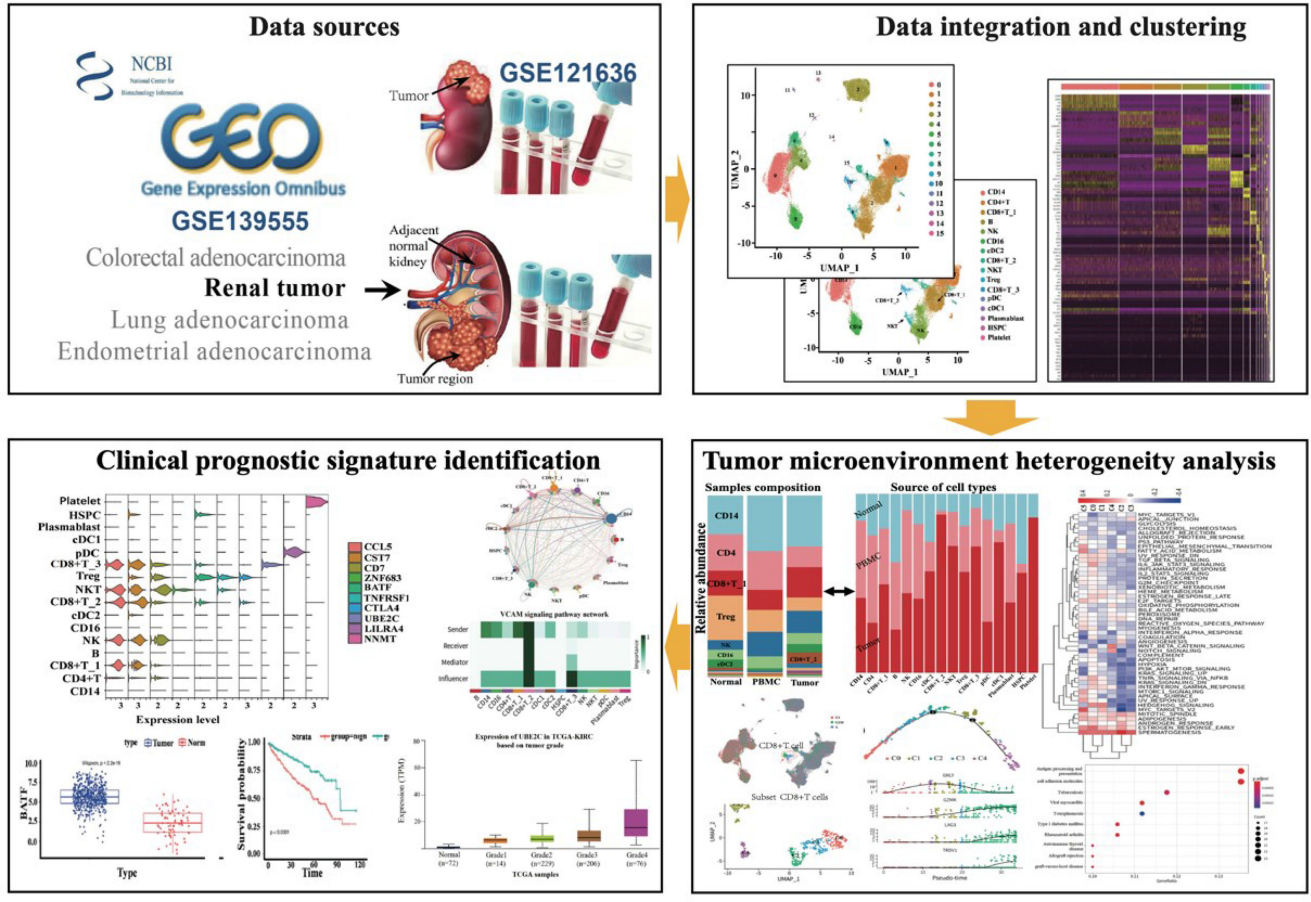


## Highlights

1. Single-cell RNA-seq decodes the signature of the ccRCC immune microenvironment
2. Dynamic changes in cell abundance and heterogeneity of cell subtypes of ccRCC
3. UBE2C expression associated with prognostic signature aids in predicting tumor progression
4. Significant decrease of B-cell populations in tumor-infiltrated immune cells

## Introduction

Clear cell renal carcinoma (ccRCC) is the most common and lethal form of renal cell carcinoma (RCC) and is responsible for more than 75% of RCC cases (1). It is a malignant tumor with multiple molecular features and a poor prognosis (2). Due to the lack of typical clinical symptoms, it is difficult to diagnose ccRCC and approximately 35% of patients had developed metastasis at the time of diagnosis (3). Studies have demonstrated that ccRCC is among the most immune and vascularly infiltrated cancer types (4). Hence, understanding tumor immune microenvironments (TMEs) is critical for identifying immune modifiers of cancer progression and developing cancer immunotherapies. For example, immune checkpoint blockade therapy and combination regimens have significantly increased survival in patients with ccRCC (5). The infiltrating CD4+ T cells can regulate the proliferation of RCC by modulating the TGFβ1/YBX1/HIF2α signals (6). However, major challenges remain, including lack of reliable predictive biomarkers and identification of more immunotherapeutic targets.

Recent applications of single-cell RNA sequencing (scRNA-seq) in dissecting TME have brought important insights into the biology of tumor-infiltrating immune cells, including their heterogeneity, dynamics, and potential roles in the disease progression and response to immune checkpoint inhibitors and other immunotherapies (7–11). The tumor immunology field has focused heavily on local immune responses in the TME, yet immunity is coordinated across the tissue. For example, many myeloid cells are frequently replenished from hematopoietic precursors in the bone marrow (12), and critical T-cell priming events typically occur in lymphoid tissues (13). The localized antitumor immune response cannot exist without continuous communication with the periphery. Among these non-cancer cells, the tumor-infiltrating immune cells (TIICs) exert a central role in pro- and anti-tumorigenic processes; moreover, they have been found to be closely correlated with the clinical outcome and response to immunotherapy (6). Previous single-cell analyses of renal cell cancers were mostly focused on solid tumor to study mechanisms of intratumorally and intertumoral heterogeneity (14), tumor microenvironment immune subtypes for classification (15), as well as distinct immune characteristic between tumor and peripheral blood or normal renal tissue (16, 17). However, the conserved or specific immune response in ccRCC across the peripheral blood mononuclear cells (PBMCs) and adjacent normal tissue in addition to within the tumor also need to be dissected. Therefore, a comprehensive understanding of ccRCC holds the promise to improve personalized treatment strategies.

In this study, we integrated publicly available single-cell RNA-seq data and comprehensively analyzed the immune characterization, as well as dynamic changes in cell subtype composition and intercellular interactions across tumor tissue. Our analyses provide insight into the immune lineages in ccRCC tumors, adjacent tissue, and PBMC. Specifically activated cellular signals in tumor tissue revealed the potential relevance to tumor progression or inflammation, and this may provide vital evidence for dissecting tumor immune response mechanism. In addition, by combining with The Cancer Genome Atlas Kidney Renal Clear Cell Carcinoma (TCGA-KIRC) RNA-seq transcriptional profile and clinical data, we focused on establishing an understanding of the associations among the TME, biomarkers, and clinical outcomes. Finally, 10 unique markers are identified to be associated with patient prognosis. Notably, UBE2C, which acts as one of the critical biomarkers specifically expressed in CD8+ T_3 cells, is involved in tumor progression and has essential prognostic value. This resource provides deeper insights into ccRCC biology that will be helpful in advancing kidney cancer diagnosis and therapy.

## Results

### Single-Cell Transcriptional Landscape of ccRCC Tumor, Peripheral Blood, and Adjacent Normal Tissue

In order to elucidate the TME of human ccRCC, we downloaded public available scRNA-seq data to dissect the transcriptome heterogeneity from tumors and matched peripheral blood from three treatment-naive ccRCC patients. In parallel, the scRNA-seq data from three other individuals derived from the renal tumor, adjacent normal tissue, and peripheral blood were downloaded for integrative analyses, aiming to facilitate the identification and assessment of ccRCC-specific differences. The data collection and quality control (QC) criteria are described in *Methods*. All high-quality cells were integrated into an un-batched and comparable dataset and subjected to principal components analysis (PCA) after correction for reading depth and mitochondrial read counts. Using graph-based uniform manifold approximation and projection (UMAP), we identified 16 clusters across 75,173 cells.

**Figure 1:**
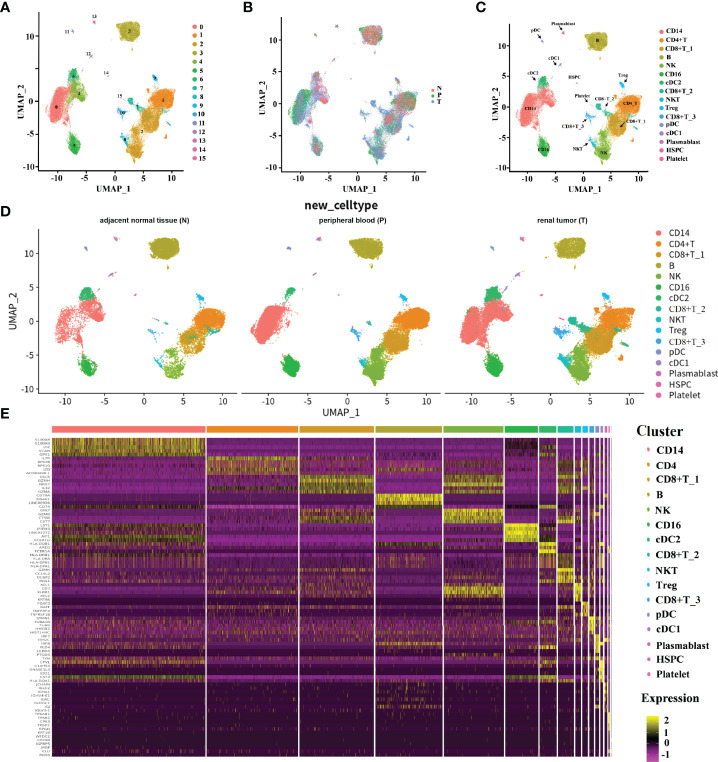
The immune landscape of patients with ccRCC at single-cell resolution. **(A–D)** UMAP embedding of transcriptional profiles from all patients and samples. Each dot represents a single cell, and colors represent clusters denoted by inferred cell type or tissue type. “N” is an abbreviation for adjacent normal tissue, “P” is for peripheral blood, and “T” is abbreviated for tumor tissue. **(E)** Heatmap of top 5 normalized expressions of markers in each cell type. Each row indicated marker genes, and columns represented cells. Yellow indicates high log-normalized expression; purple indicates low log-normalized expression.

A total of 31,093 cells originated from the tumor, 14,788 cells were normal adjacent tissue-derived, and 29,292 cells were obtained from peripheral blood (**Figures 1A, B**). We cataloged cells into 16 distinct cell lineages, including myeloid (CD14 and CD16 cells, cDC2, cDC1, and pDC cells), T cells (CD4+ T cells, Treg, CD8+ T_1, CD8+ T_2, CD8+ T_3 cells, and NKT cells), B cells (B, plasmablast cells), NK cells, HSPCs, and platelets as the common cell types (**Figure 1C**). Seurat (18) cell reference datasets, SingleR package (19), and known markers in the CellMarker database (20) were together used for this cell-type annotation. It is obvious to see that the distribution of the cells from different tissue types of ccRCC is similar, but the abundance of certain subclusters is different, while the original study reported that the cells derived from renal tumors, matched peripheral blood, and healthy normal kidneys were enriched in distinct clusters. This illustrated that the immune response in pathology is different from the healthy condition. The relative proportion of cell types comprised by tissue type is illustrated in **Figure 1D**. The top five markers of the main cell lineages were visualized as a heatmap (**Figure 1E**).

### Cell-Type Differences and Hallmark Signatures in ccRCC

**Figure 2:**
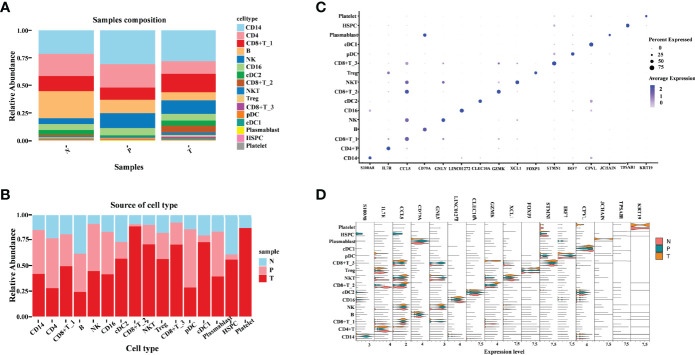
Illustration of the heterogeneity of ccRCC tumor immune environment in different tissue types. **(A)** Cell-type abundance in each tissue type. Each bar corresponds to one tissue type, colored according to the cell types. **(B)** Sample distribution in each cluster. Each bar corresponds to one cell-type cluster, colored according to the samples. **(C)** Dot plot of canonical cell-type markers of 16 major cell types. The circle size indicates the percentage of genes expressed in a cell. The darker the color indicates a higher average expression level. **(D)** Violin plot of canonical cell-type markers of 16 major cell types in different tissue types.

To be more comprehensive and intuitive in observing changes in cell composition in different groupings, cell components were visualized in the form of a bar plot in the form of cell types and samples (**Figures 2A, B**). The cell types contained in different samples were almost identical but the abundance of cells in each cell type was varied. Among the three groups, the abundance of CD14 cell clusters is the most of all the cell clusters, which indicates a vital role of CD14 in tumor immune response. We observed a great decrease of CD4+ T cells and B cells within tumors and adjacent normal kidneys relative to peripheral blood (**Figure 2A**). This is similar to the findings of the original study by Borcherding et al. They observed a decreased CD4+ T cells and B cells within healthy normal kidneys or tumors relative to peripheral blood (17). In addition, we also noticed an increase in three subtypes of CD8+ T cells (CD8+ T_1, CD8+ T_2, and CD8+ T_3), and NKT cells in tumors relative to adjacent normal tissue and peripheral blood. The increasing tendency is similar to the decreasing tendency of CD4+ T cells and B cells. In the original study, the authors also revealed that the trends of increased CD8+ and decreased CD4+ T cells were similar, which is performed by immunohistochemistry on paired normal and tumor tissue. cDC1 and cDC2 cell abundance shows a clear decreased tendency from tumor versus adjacent normal tissue and peripheral blood derived from ccRCC patient samples, indicating the activation of adaptive immune responses. The relative abundance of NK cells, CD16 cells, and plasmablast cells in the tumor was comparable to that in peripheral blood but higher than that in adjacent normal tissue. Notably, HSPC cells were significantly enriched in tumor comparison with adjacent normal tissue, and the abundance of HSPC in peripheral blood is much lower. Furthermore, we notice that platelet cells are almost only in the tumor and adjacent normal tissues, and the platelet cells were highly enriched in tumor tissues (**Figure 2B**); this suggested an important function of platelets in TME and the central component during the development of tumors. The above result revealed that the tumor microenvironment of ccRCC is highly heterogeneous. A deep understanding of the tumor microenvironment, especially the characteristics of tumor-infiltrating immune cells, is crucial for exploring the key regulatory molecules of tumor development.

Next, we focused on the transcriptomic features of each major cell type. Specific marker genes of each cell cluster were obtained according to their gene profiles, picking the marker gene with the best specific indicative effect (**Figure 2C**). To explore the existence of differential expressions of these marker genes in different groups, a violin pagination map was made based on the sample source (**Figure 2D**). Most of these markers’ relative expression in tumors was comparable to that in peripheral blood and adjacent normal tissue; the differences in these gene expression patterns were only exhibited in distinct subtypes. The following three genes are obviously different in their expression: (1) S100A8 (S100 Calcium Binding Protein A8), which plays a prominent role in the regulation of inflammatory processes and immune response. It was highly enriched in CD14 cells derived from peripheral blood. The unique high expression level of S100A8 in the CD14 cell cluster indicates that the CD14 cells may play pro-inflammatory and anti-tumor roles in ccRCC. (2) JCHAIN (Joining Chain of Multimeric IgA and IgM), which is almost only detected in tumor-derived plasmablast cells. One of the JCHAIN-related pathways is cell surface interactions at the vascular wall; it indicated that the role of plasmablast is closely related to tumor angiogenesis. (3) The third gene was KRT19 (Keratin 19), whose related pathways are embryonic and induced pluripotent stem cell differentiation pathways and lineage-specific markers. In this study, we found that it was detected in platelet cells derived from tumor and adjacent tissue. These marker genes emphasized that the transcriptional features of various immune cells derived from different tissue types are specificity, further demonstrating the immune response heterogeneous in the tumor, matched adjacent normal tissue, and peripheral blood.

### CD8+ T_3 Cluster Associated With the Cell Cycling Process

**Figure 3:**
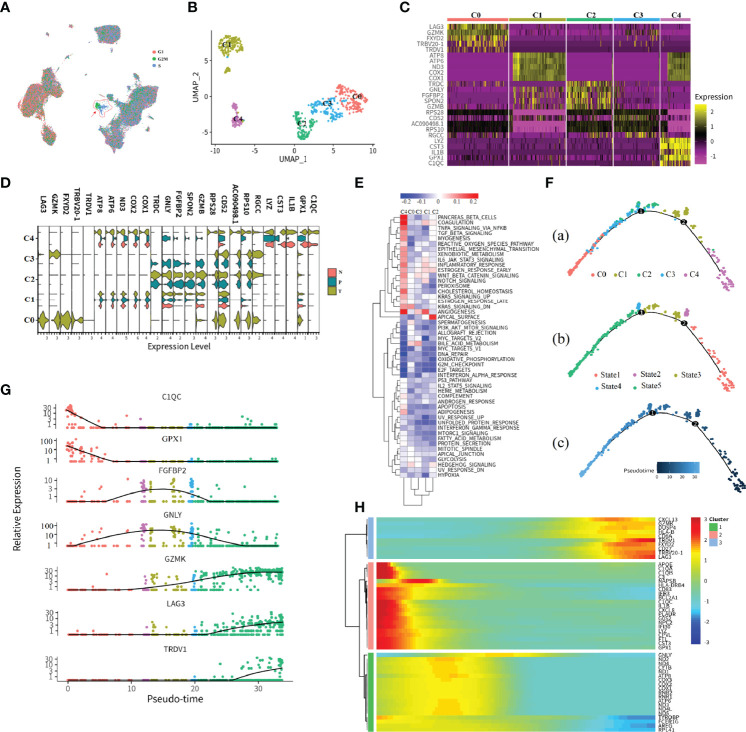
Subtypes and development trajectory of CD8+ T_3 cells. **(A, B)** UMAP visualization of all cell types **(A)** or CD8+ T_3 subtype **(B)**. Each dot corresponds to a single cell, colored according to cell cycle state or subtype. **(C)** Heatmap of selected markers for the CD8+ T_3 subcluster. **(D)** Violin plot of the CD8+ T_3 subtype in different tissue types. **(E)** Hallmark enrichment visualization of each subtype. The redder color indicates an upregulated pathway activity; the bluer color means downregulated pathway activity. **(F–H)** Illustration of CD8+ T_3 cell development trajectory inferred by monocle2, and canonical markers of each state were selected to visualize the cell development. Heatmap showing relative expressions of canonical markers of CD8+ T_3 cells along inferred trajectories. The red and blue colors correspond to the relative gene expression level.

Clustering of cells revealed three distinct CD8+ T subtypes with relative transcriptional specific features, and CD8+ T_3 subtypes highly exhibited cell cycle association characteristics that were mainly in S or G2M phases (**Figure 3A**). To further understand the characteristics of the tissue-specific distribution of CD8+ T_3 subclusters, CD8+ T_3 cells were sub-clustered into five distinct clusters (**Figure 3B**). The top 5 marker genes in each subgroup were selected to visualize the specific gene expression pattern (**Figures 3C, D**). The expression of these marker genes in different tissues showed high heterogeneity. Tissue-infiltrating CD8+ T cells (both tumor and adjacent normal tissue) comprised the majority of C1 and C4. Clusters C2 and C3 comprised tumor and peripheral blood cells, and cluster C0 cells were only derived from renal tumor cells. Going from left to right across the *x*-axis of the UMAP, there is a change in tissue-specific contribution starting from peripheral blood, tumor, and adjacent normal tissue to the tumor, which may represent the process of tissue infiltration CD8+ T3 cells. Previous studies revealed that the proliferation of CD8+ T cells is an important surrogate marker of the antitumor immune response (17, 21). Furthermore, we found that the C0 subcluster has high expression of inhibitory checkpoints, including LAG3, CTLA4, GZMK, and PDCD1. Since these molecules are markers of T-cell exhaustion, these data indicated that C0 subclusters were exhausted CD8+ T cells. At present, PD-1/PD-L1 and CTLA4 are the most popular targets of immunotherapy. Since the expression of LAG3, GZMK, FXYD2, and TRBV20-1 was higher than that of PD-1 in the exhausted T-cell subcluster, our data revealed that LGA3, GZMK, FXYD2, and TRBV20-1 may serve as potential targets for ccRCC immunotherapy.

In order to assess possible functional differences based on these subclusters in CD8+ T3 cells, we performed gene set variation analysis (GSVA) (**Figure 3E**). As expected, based on the process features of tissue infiltration of CD8+ T3 cells (**Figure 3D**), *PANCREAS_BETA_CELLS*, *COAGULATION*, and *ANGIOGENESIS* pathways were upregulated in C4 and C1 clusters (**Figure 3E**), and the *APICAL_SURFACE* signal pathway and the *BILE_ACID_METABOLISM* pathway are upregulated in C2 and C3 clusters (**Figure 3E**), respectively. Cluster C0 showed upregulated activity of the *KRAS_SIGNALING_DN* pathway (**Figure 3E**). These specific pathways in different subsets reflected the characteristics of tumor immune infiltration and response processes. For example, the C2 and C3 clusters exhibited obviously metabolic re-program characteristics, while C1 and C4 demonstrated a strong immune stress response of immune cells no matter which tissue they were derived from in tumor patients, and cluster C1 indicated that the expression of immune checkpoint inhibitors may be associated with the KRAS pathway.

In order to examine gene expression patterns across distinct subclusters in CD8+ T_3 cells, we utilized monocle2 (22) to build branched structures among subclusters, inferring the developmental trajectory of CD8+ T_3 clusters (**Figure 3F**). We identified one major curve with the origin in C4; cluster C0, which represented an exhausted T-cell cluster, was inferred as the end state of the differentiation trajectory; C1 and C2 cells were located between these two end states. Afterwards, genes were selected based on biological functions or immune response features to prove the inferring, and the result is consistent with the inferred trajectory (**Figure 3G**). For example, LAG3, which is an immune checkpoint inhibitor that represented exhausted T cells, showed high expression at the late pseudotime. In order to ensure the accuracy performance of inferring trajectory, we also performed trajectory inference by the dyno package (23), which is a benchmark of trajectory inferring methods for cellular ordering, topology, scalability, and usability. The top three good performances inferring results of trajectory show high consistency with the predicted results of monocle2 (24) (**Supplementary Figures 1A–3A**). Lastly, the top 50 differentially expressed genes (DEGs) were selected to visualize the features of gene expression in developmental trajectory in a heatmap (**Figure 3H**). The expression pattern of these genes ordered by pseudotime was examined by the top three methods with good performance (**Supplementary Figures 1B–3B**).

### Hallmark Signatures and Metabolism Disturbance of the CD8+ T_2 Cluster

**Figure 4:**
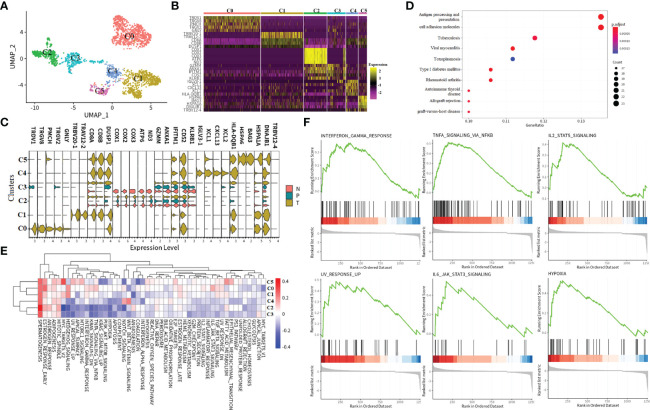
Subtypes and immune response characters of CD8+ T_2 cells. **(A)** UMAP visualization of the CD8+ T_2 subtype. Each dot corresponds to a single cell, colored according to cell subtype. **(B, C)**Heatmap of selected markers for CD8+ T_2 subcluster and visualization of the expression level of selected markers in each tissue type. “N” indicates tumor in matched adjacent normal tissue, “T” means renal tumor tissue, and “P” represents peripheral blood. **(D)** KEGG pathway enrichment analysis of the CD8+ T_2 subtype. The points size indicates the counts of genes enriched in a specific pathway, and the color means the statistical significance. **(E)** GSVA enrichment visualization of the CD8+ T_2 subtype. The red color indicates that the specific pathway is upregulated in a subtype, and the blue color means the pathway is downregulated. **(F)** GSEA pathway enrichment visualization of differentially expressed genes in tumor tissue compared to adjacent normal tissue in the CD8+ T_2 subtype. All six pathways are upregulated in renal tumor tissue.

The previous observation showed that the majority of cells in the CD8+ T_2 subcluster were derived from tumor tissue (**Figure 2B**), indicating that it is the most infiltrating CD8+ T-cell subset in tumor tissue. Hence, we next focused on the transcriptomic features of the CD8+ T_2 subcluster. Across CD8+ T_2 cells, sub-clustering found six distinct clusters that were labeled as C0 to C5 (**Figures 4A, B**), and the expression patterns of specific marker genes in distinct subclusters were further examined (**Figures 4B, C**). Results exhibited tissue-specific distribution, with the majority of tumor-infiltrating CD8+ T cells in C0, C1, C4, and C5. In contrast, both C2 and C3 clusters were composed of renal tumors, in matched adjacent normal parenchyma, and peripheral blood. The expression patterns of marker genes of different tissues were visualized as violin plots (**Figure 4C**). Marker genes in tumor-infiltrating CD8+ T cells could be mainly classified into three types: (1) T-cell receptor-associated genes, such as TRDV1, TRGV2, TRGV8, and TRBV20-1; (2) chemokines and cytokines, such as CXCL13, XCL1, and XCL2; and (3) stressful stimuli genes, such as BAG3, HSPA6, and HPPA1A. The above genes played a key role in tumor growth or inflammation. Notably, in the C3 cluster, marker genes like COX1, COX2, COX3, ATP6, and ND3 were only detected in adjacent normal tissue, and these genes play important roles in the mitochondrial oxidative respiratory chain. The finding indicated that mitochondrial oxidative stress is strongly linked to immune responses in tumor progression, and it may contribute to exploring the different immune responses in the tumor, adjacent normal tissue, and PBMC.

In order to assess possible functional differences based on these subclusters, we performed the KEGG pathway and GSVA analysis (**Figures 4D, E**). The KEGG enrichment results show that these marker genes in the CD8+ T_2 cluster are essential in interfering with antigen presentation, apoptosis, and host immune system response (**Figure 4D**). The MITOTIC SPINDLE, ADIPOGENESIS, ANDROGEN_RESPONSE, ESTROGEN RESPONSE, and SPERMATOGENESIS pathways all upregulated in CD8+ T_2 subclusters from C0 to C5 (**Figure 4E**). We noticed that the activities in certain pathways exhibited two patterns: in C2 and C3 subclusters, the pathway activities are downregulated, while in C0, C1, C4, and C5, the activities of pathways are upregulated. As the cells in C0, C1, C4, and C5 are all tumor-infiltrating, we performed gene set analysis on the genes in C0, C1, C4, and C5 compared with genes in clusters C2 and C3. The results revealed that the INTERFERON_GAMMA_RESPONSE pathway, the IL2_STAT5_SIGNALING pathway, the TNFA SIGNALING VIA NKFB pathway, the UV_RESPONSE_UP, IL6_JAK_STAT3_SIGNALING pathway, and the HYPOXIA pathway were upregulated in tumor-infiltrating cells (**Figure 4F**). The six hallmark signals not only produce more parsimonious but equivalent results, avoiding the problem of gene set redundancy and over-representation altogether, but also provide candidate pathways that play a vital role in ccRCC tumor tissue.

### Relationship Between Prognostic Features and Cell Type-Specific Marker Genes in ccRCC

To assess the clinical significance of expression of a given gene set, we utilized RSEM normalized log2 bulk RNA-seq expression data available from TCGA through the Firehose pipeline hosted by the Broad Institute (25). We utilized clinical and outcomes data available through the TCGA project website. From these resources, we filtered our analysis by patients who underwent testing for a renal tumor or in matched adjacent tissue. We then scaled and centered the log2 bulk RNA-seq data for the patients, and used this dataset for gene set enrichment analysis.

**Figure 5:**
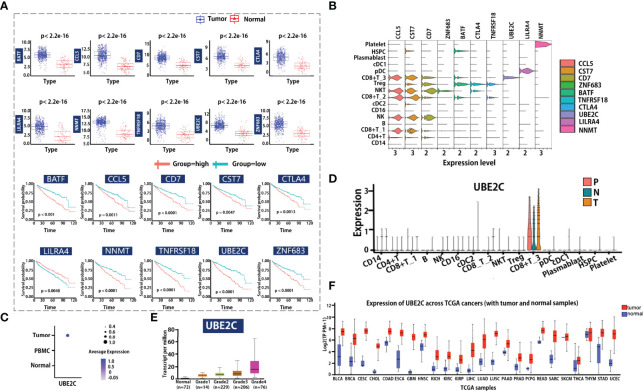
Clinical outcome correlation analysis of cellular composition and gene expression. **(A, B)** Marker genes identified by bulk RNA-seq data associated with scRNA-seq data. Boxplot visualized the gene expression level of genes in the tumor or adjacent normal tissue group **(A left)**. Each dot represents a candidate gene; the blue and red color means tumor or matched adjacent normal tissue group. Survival analysis for candidate markers of each cell type in ccRCC **(A)** right. The red and light blue lines indicate the groups with high or low gene expression. Violin plot shows the candidate markers’ expression profile in all major cell types. **(C, D)** Dot plot shows the UBE2C gene expression level in different tissue types or cell types. N indicates tumor in matched adjacent normal tissue, T means renal tumor tissue, and P represents peripheral blood. **(E)** Visualization of the association relationship of the expression level of UBE2C with ccRCC tumor grades. **(F)** Comparing the expression of UBE2C in tumor tissue and adjacent normal tissue across pan-cancer.

To determine if these transcriptional differences led to functional differences in tumor response, we investigated whether gene signatures were associated with prognostic values. Using the TCGA dataset for ccRCC, a total of 899 DEGs between tumor and adjacent normal tissue were identified, 281 survival-associated DEGs were obtained after using univariate Cox proportional hazard regression to evaluate the association between the expression of DEGs and patient overall survival (OS). In addition, by overlapping prognostic-associated DEGs with the top-ranked marker genes in 16 major subclusters at single-cell resolution, 10 cell type-specific marker genes that were associated with prognosis were obtained. The transcriptional features of the 10 markers, including expression patterns in tumor or in matched adjacent normal tissue, as well as the prognostic signatures, are visualized in **Figure 5A**.

Furthermore, we compared these gene expression patterns at single-cell resolution (**Figure 5B**), and the result showed that the expression of these genes in different immune cells is totally distinct. For example, ZNF683 is mainly expressed in NKT cells, while BATF, TNFRSF18, and CTLA4 are mainly expressed in Treg cells. The UBE2C gene is highly enriched in the CD8+ T_3 cluster (which is associated with cell cycle progress), and LILRA4 is mainly enriched in pDC cells. We utilized clinical data from TCGA to explore whether the expression of these genes is associated with tumor grade. Notably, the cell cycle G2/M phase gene UBE2C is specifically expressed in tumor-infiltrating cells (**Figure 5C**), and the expression levels of the UBE2C gene in the tumor, adjacent tumor, and peripheral blood are basically the same (**Figure 5D**). We also observed that the UBE2C gene expression was associated with increasing histological grades (**Figure 5E**), indicating that it may be a potential novel target for tumor progression prediction. Lastly, we compare the gene expression of UBE2C across TCGA cancers (**Figure 5F**), results revealed that the gene expression is significantly different between tumor and normal. This indicates that the UBE2C may be a good biomarker for patients on clinical application.

### Cell–Cell Communication Diversity in Distinct Tissues in ccRCC

**Figure 6:**
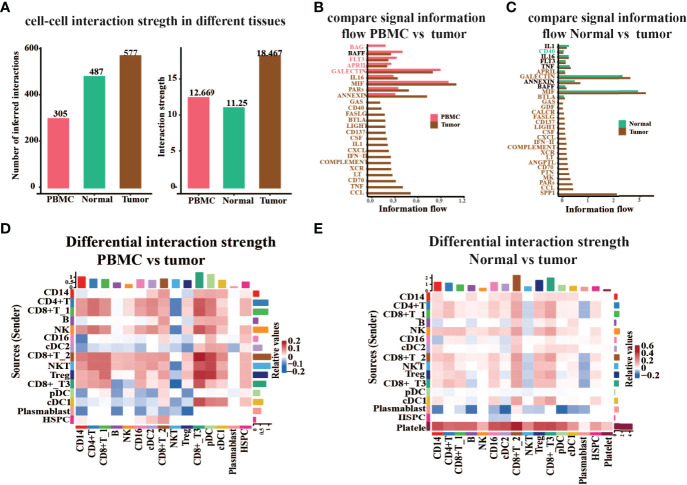
Global analysis cell–cell interaction features in different tissue types. **(A)** The number or strength of the interaction among all major cell types in peripheral blood (PBMC), tumor-adjacent normal tissue (Normal), and renal tumor tissue (Tumor). **(B, C)** Comparison of the signal information flow in tumor compared to PBMC **(B)** or normal **(C)**. **(D, E)** Heatmap of different interaction strengths in tumor compared to PBMC **(D)** or Normal **(E)**. The vertical axis is the cell sending the signal, the horizontal axis is the cell receiving the signal, the shades of color on the heatmap represent the relative signal strength, and the bars on the top and right represent the cumulative intensity on the horizontal and vertical axes.

To better understand global communications among cells in tumor progression, accurate representation of cell–cell signaling links and global analyses of those links were required. We integrated cell–cell communications using the CellChat (26) R package across all cell types in ccRCC (**Figure 6**). As there were no cells detected in platelet cell groups derived from PBMC, cell–cell communication analysis only focused on the other 15 major cell types. We first examined the overall patterns of communication across all cell populations, and statistical analysis of the strength of cell interactions was performed (**Figure 6A**). Results revealed that the cell–cell interaction strength in tumor tissue is obviously higher than that in PBMC and adjacent normal tissue. Next, we compared the signal information flow for each signaling pathway between PBMC and tumor tissue, or adjacent normal and tumor tissue (**Figures 6B, C**). The information flow for a given signaling pathway is defined by the sum of communication probability among all pairs of cell groups in the inferred network. We found that some pathways, including BAFF, IL1, IL16, FLT3, TNF, and ANNEXIN, maintain a similar flow between the tissue conditions (black in **Figures 6B, C**). We interpret that these pathways are equally important in the tumor progression or immune response in both tissues. In contrast, other pathways prominently change their information flow at PBMC or normal as compared to tumor tissue: (i) turn off (BAG), (ii) decrease (such as BAFF, FLT3, and IL1), (iii) turn on (such as GAS and LIGHT), or (iv) increase (such as MIF, PARs, ANNEXIN, and IL16).

Moreover, we studied the detailed interaction strength across all cell types in different groups (**Figures 6D, E**). Results once again demonstrated that the communication strength of cells in distinct tissues is significantly altered. The cell–cell interaction strength in tumor tissue experienced an obvious increase in CD8+ T_2 cells, CD8+ T_3 cells, NKT cells, and Treg as compared to PBMC (**Figure 6D**). When we compare the interaction strength between normal adjacent tissue to tumor tissue, we noticed that the strength in tumors almost increased. However, the signal information sent from plasmablast to other cells was decreased and the strength of interaction from platelet cells to other cell types was increased in tumors as compared to normal adjacent tissue (**Figure 6E**). Hence, these results revealed that cell–cell communication is highly associated with pathophysiological characteristics of the tumor microenvironment.

### Discovering Major Signaling Changes in Response to ccRCC

**Figure 7:**
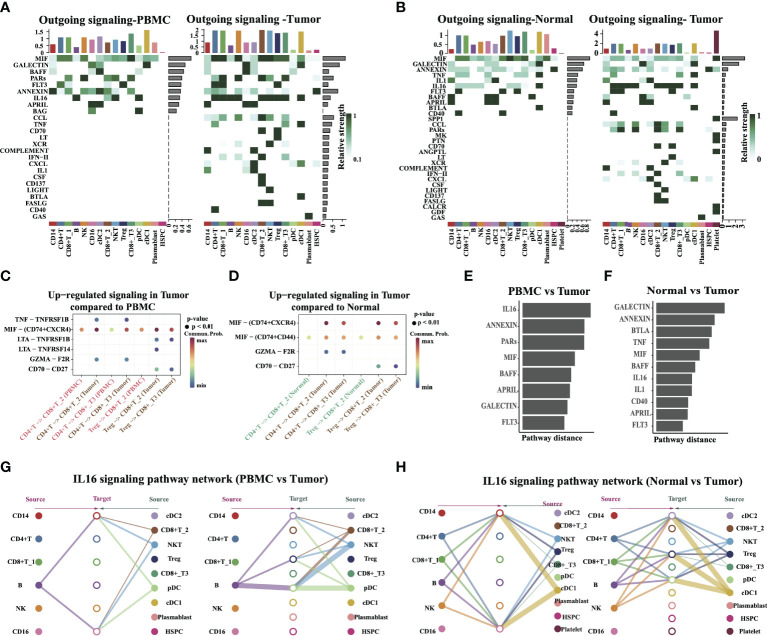
Identify signal patterns in major cell types. **(A, B**) Outgoing communication patterns identification in different tissue types. The color indicates the relative strength of the signal among cells. The horizontal axis is the cell type, and the vertical axis is the pathway. Bars and heatmaps above and to the right correspond to cumulative signal strength. **(C, D)** Comparison of the significant ligand–receptor pairs between PBMC vs. Tumor, and Normal vs. Tumor, which contributed to the signaling from CD4+ T/Tregs to CD8+ T cells (CD8+ T_2, CD8+ T_3). Dot color reflects communication probabilities and dot size represents computed p-values. Empty space means the communication probability is zero. **(E, F)** The overlapping signaling pathways in PBMC vs. Tumor, and Normal vs. Tumor were ranked based on their pairwise Euclidean distance in the shared two-dimensional manifold. A large distance implies a larger difference. **(G, H)** Hierarchical plot showing the inferred intercellular communication network of the IL16 signaling pathway in PBMC vs. Tumor and Normal vs. Tumor, respectively. The edge width represents the communication probability.

Intercellular connections are an important pathway for cell–cell crosstalk. Such crosstalk in cells is critical for informing diverse cellular decisions, including decisions to activate the cell cycle or programmed cell death, undergo migration, or differentiate along the lineage. In humans, cell–cell crosstalk is mediated through ligand–receptor signaling pathways or secretion/uptake of exosome-transmitting information across the surrounding intercellular environment. Hence, we studied the detailed changes in the outgoing signaling across all significant pathways using pattern recognition analysis. There are 25 and 30 signaling pathways with differential strength in tumors compared to PBMC or normal tissue, separately (**Figures 7A, B**), including MIF, GALECTIN, FLT3, IL16, CCL, and MK pathways. We found that the accumulated effect strength of platelet cells obviously increased in tumor tissue compared with PBMC or normal tissue. Notably, the SPP1 signal is turned on in tumor tissue, and the accumulated effect strength of it in the tumor is obviously higher than other pathways. This suggests that SPP1 is an important pathway that may be associated with tumor progression.

Next, we inferred intercellular communication networks for the tumor as compared to PBMC or adjacent normal tissue separately. Six pathways were specifically active in tumors compared to PBMC, including known inflammatory signals MIF, TNF, and IL16, suggesting that these pathways might critically contribute to tumor progression. Specific to MIF signaling in tumor tissue, CellChat identified ligand MIF and its multi-subunit receptor CD74+CXCR4 as the most significant signaling, contributing to the communication from CD4+ T cells to CD8+ T2 and CD8+ T3 cells (**Figures 7C, D**). This is in agreement with a reported experiment finding. That study reported that CD74 and CXCR4 are upregulated in renal cells in diseased kidneys and MIF activation of CD74 in kidney cells promotes an inflammatory response (27). Ligand TNF and its receptor TNFRSF1B were found to act as major signaling from CD4+ T cells to CD8+ T2/T3 cells, and the ability of the TNF–TNFRSF1B pair to kill tumor cells *in vitro* has been reported before (28). Ligand CD70 and its receptor CD27 were also found to be active in tumors, in particular, for the signaling from Treg to CD8+ T2 and CD8+ T3 (**Figures 7C, D**). This reveals the dynamic changes in the levels and patterns of ligand–receptor expression in different tissue or tumor progression conditions.

By computing the Euclidean distance between any pair of the shared signaling pathways, we observed a large distance for signaling pathways like IL16, ANNEXIN, GALECTIN, BTLA, TNF, and MIF, suggesting that these pathways exhibit significantly different communication network architectures in tumors compared to PBMC or adjacent normal tissue (**Figures 7E, F**). The signaling pathways also show relatively small distances in tumors as compared to normal adjacent tissue, including CD40, APRIL, and FLT3. This indicates communication network architectures for these overlapping pathways in both tumor and normal tissues. A closer look at the IL16 pathway (**Figures 7G, H**) shows its high signaling redundancy (i.e., multiple signaling sources) and high target promiscuity. The latter finding indicated that certain pathways have highly conserved signaling architecture (i.e., high degree of redundancy), which is largely independent of the specific cellular composition of the tissue. Taken together, the results provided insights into the heterogeneity of cell–cell interactions from different locations within the tumor, and it may reveal how various tissue microenvironments influence which cell–cell interactions occur.

## Discussion

With the improvement of understanding of how immunotherapies work, the phenotypic and functional profile of immune cells in the TME is now well known to influence prognosis and disease outcome. The latest advances in immunotherapy are completely changing the pattern of clinical immuno-oncology and are significantly improving the survival rate of patients with various cancers (29). Although the single-cell expression profile of ccRCC was previously reported (17), the original study mainly focuses on the distinct immune characteristic of tumor and peripheral blood or normal renal tissue. In this study, we integrated publicly available single-cell RNA-seq data and comprehensively analyzed the immune characterization. Compared with adjacent normal tissue and PBMC, CD8+ T2 and CD8+ T3 cells were significantly tumor-infiltrating cell clusters, which were cell cycle or T-cell exhaustion-associated cells, indicating well-functioning immunosurveillance in tumor tissue of ccRCC (30–32). Consistent with this finding, enrichment of CD8+ T (CD8+ T1, CD8+ T2, and CD8+ T3) cells and DCs (cDC1 and cDC2) in tumor tissue conferred enhanced immune activation and recruitment of antitumor effector cells (32, 33), while the abundance of B cells was fewer in tumor tissue compared to adjacent normal tissue and PBMC. B cell-mediated antibody production can lead to the killing of tumor cells through the complement cascade activation, phagocytosis by macrophages, and activation of the tumor-killing activity of NK (34). The decreased population of B cells may suggest an increased pro-tumoral activity. The different abundance of specific cell types in distinct tissues revealed the immune response association and heterogeneity in the tumor, adjacent normal tissue, and PBMC. The results will facilitate the understanding of intratumor heterogeneity and the immune microenvironment complexity in the ccRCC.

As critical players in tumor immunity, CD8+ T cells can directly recognize and kill cancer cells upon recognition of neoantigens. In addition, it also responds to various cues to tune their developmental lineages and states. In this study, we identified three subclusters of CD8+ T cells. We revealed that CD8+ T_3 cluster cells, which mainly stay on cell cycle G2/M and S1 phases, showed exhaustion characterization in ccRCC. T-cell exhaustion was previously characterized as a loss of proliferative capacity and high levels of PD-1 and LAG3 expression in human tumors (28, 35, 36). We identified several markers specifically expressed in the C0 cluster that was tumor-infiltrated, such as checkpoint inhibitor (LAG3), cytotoxicity-associated genes (GZMK), and oncogenic driver gene (FXYD2), further indicating that these CD8+ T cells were exhausted. This finding is consistent with the original study (17). In addition, we noticed that the expression of LAG3 and GZMK is higher than that of immune inhibitors, such as PD-L1, TIM3, and CTLA4, in the CD8+ T_3 cluster; thus, LAG3 or GZMK may be a better target for tumor immunotherapy in ccRCC.

A total of 10 candidate biomarkers were obtained by combining analysis with TCGA-KIRC bulk RNA-seq data. Some of them have been mentioned in hematological malignancies or solid tumors and affect tumor progression (35, 37–41). Previous studies may report that some of them were associated with prognostic signature (36, 42–44), but they all focus on gene expression level or epigenetic modification. In this study, we identified several cell type-specific expressed biomarkers, including CCL5, BATF, and CTLA4 at a single-cell resolution, whose good clinical prognostic signature may be highly associated with specific immune cell types, like CD8+ T cells and Tregs. Lin et al. (45) reported that CCL5 was highly expressed in breast cancer lymph node metastasis and that CCR5–CCL5 interaction promotes cancer cell migration under hypoxic conditions. Zhang et al. (46) reported that CCL5 deficiency delayed tumor growth and metastasis *via* facilitating CD8+ T cells to accumulate into the tumor site. NNMT was identified as one clear cell renal cell carcinoma (ccRCC)-associated gene (47), and it induces the proliferation and invasion of squamous cell carcinoma cells (48); Tang et al. (47) demonstrated the crucial role of NNMT in the promotion of cellular invasion in ccRCC cell lines. Therefore, the high expression of NNMT in ccRCC was linked to poor prognosis, further suggesting that it may be a potential biomarker for worse prognosis. We also found that CTLA4 was upregulated in CD8+ T_2 and Treg cells in ccRCC tissues and closely related to the disease progression as well as a poor prognosis. This is consistent with the previous study result that Tregs can inhibit the activation of CD8+ T cells through CTLA4, triggering tumor immunosuppression (49). In addition, we found that CTLA4 was markedly correlated with multiple immune checkpoints, which suggested that ccRCC patients with high expressed CTLA4 may benefit more from immune checkpoint blockade combined therapy. Notably, we identified UBE2C, the most important biomarker that is specifically expressed in exhausted CD8+ T cells, as being associated with clinical factors including TNM stage, gender, and pathological stage. Higher UBE2C expression predicted shorter OS and progression-free survival. This is consistent with the previous study that UBE2C is an important gene in ccRCC and is essential to the proliferation and migration of ccRCC (50). Strikingly, we noticed that the expression of UBE2C in CD8+ T_3 cluster cells is almost identical, regardless of origin (tumor tissue, adjacent normal tissue, or PBMC in ccRCC). Hence, we concluded that UBE2C may be a critical factor for predicting the prognosis of ccRCC patients and that the detected expression level of UBE2C from PBMC may contribute to predicting tumor progression and aid immunotherapy in ccRCC.

## Conclusion

In conclusion, this study comprehensively compares the transcriptomic signature in different regions of ccRCC, and it unmasked the conserved and specifically activated signals among tumor tissue, adjacent normal tissue, and PBMC in ccRCC. The biomarkers identified uncovered the correlations between gene expression, survival outcomes, and immune cell-type components, aiding in the development of more effective immunotherapy strategies for ccRCC.

## Methods

### GEO Dataset Acquirement

All samples were obtained from the National Center for Biotechnology Information GEO dataset. GSE121636 (17) performed single-cell sequencing on the peripheral blood and tumor-infiltrating cells of three patients with renal clear cell carcinoma. GSE139555 (51) sequenced the peripheral blood, adjacent tissues, and tumor tissues of three patients who were sick of renal cell carcinoma. We divided all patients into three groups according to their origin tissue (PBMC: peripheral blood; T: renal tumor; N: normal adjacent tissue).

### Single-Cell Data Processing

Raw data were converted into a Seurat object by the R package Seurat (v 3.1.2) (18). Cells whose percentage of ribosomes or percentage of mitochondria are less than 15 or single cells with less than 500 genes detected were considered low-quality cells and were removed. In order to eliminate potential doublets, single cells with over 4,000 genes detected were also filtered out. Finally, 75,173 single cells remained, and they were applied in downstream analyses.

After quality control, the Seurat object was normalized by the function SCTransform of the Seurat package. Since samples from six patients were processed and sequenced in batches, their origin tissue was used to remove the potential batch effect. In this progress, the top 2,000 variable genes were used to create potential anchors with the FindIntegrationAnchors function of Seurat. Subsequently, the Harmony function was used to integrate data and merge a new matrix with 2,000 features, in which potential batch effect was regressed out.

To reduce the dimensionality of the scRNA-Seq dataset, PCA was performed on an integrated data matrix. With the elbowplot function of Seurat, the top 30 PCs were used to perform the downstream analysis. The main cell clusters were identified with the FindClusters function offered by Seurat, with resolution set as default (res = 0.8), and then they were visualized with 2D UMAP plots (52). Conventional markers described were used to categorize every cell into a known biological cell type. Firstly, all cells were clustered into twenty-four major clusters and further clustered into sixteen clusters in a previous annotation by Azimuth, SingleR (19), and CellMarker database (20). All details regarding the Seurat analyses performed in this work can be found in the website tutorial (https://satijalab.org/seurat/v3.0/pbmc3k_tutorial.html).

### Cell–Cell Communication Analysis

The CellChat v1.1.3 software (26) was used to infer cell–cell communication based on ligand–receptor interaction with default parameters. For each ligand–receptor pair, only the secreted signaling interaction category was considered for downstream analysis. We filtered out the cell–cell communication if there are fewer than 15 cells in certain cell groups. The statistical significance of communication probability values was assessed by a permutation test. *p* < 0.05 was considered statistically significant.

### Marker Gene Selection Is Specific to Clusters

For pairwise comparisons between clusters, we manually calculated the log2 fold change (log_2_FC) between each cluster using the Seurat FindMarkers function. Genes were required to be expressed in >10% of cells with each of the sixteen cell clusters. Genes were selected as marker genes based on the statistical threshold [log2FC > 0.25, adjusted *p*-value (Bonferroni) < 0.01]. The top 5 genes of each cluster were visualized with heatmap plots.

### Data Collection and Code Availability Statement

The datasets analyzed during the current study are available in the TCGA, TCGA-KIRC, and GEO repository (GSE121636 and GSE139555). The data and code are available in GitHub (https://github.com/Wang-biolab/scRNA-ccRCC/). Transcriptome RNA-sequencing data of KIRC were downloaded by the R package RTCGA.rnaseq. There were 534 cases of KIRC tissues and 72 cases of normal tissues. The clinical information and demographic data were also obtained by the R package RTCGA.clinical.

### Differentially Expressed Genes

DEGs between KIRC tissues and normal tissues were preliminarily screened *via* the R software limma package and edgeR package. DEGs were determined with the following cutoff value: false discovery rate (FDR) = 0.01, log_2_|fold change| = 3.

### Survival Analysis

The OS was involved as the endpoint and index for the prognostic outcome. For DEGs, the survival R package was applied to screen the survival-associated DEGs by univariate Cox analysis, with a *p*-value < 0.01 based on the log-rank test. Kaplan–Meier curves were generated to illustrate the relationship between patients’ OS and gene expression levels of DEGs.

### Pathway and Functional Annotation

A gene functional enrichment analysis was performed based on the marker genes in each cell cluster using KEGG pathway enrichment analysis. These DEGs were loaded into cluster profiles for the GO and KEGG pathway enrichment analysis (53). Pathways for which the adjusted *p*-value was less than 0.05 were considered significantly enriched.

Gene set enrichment analysis (GSEA) algorithm was implemented to evaluate the relative activation status between gene expression within clusters and sets of genes of known biological significance. Gene sets used for analysis were derived from the C7 (immunological gene sets) database available through the MSigDB Collections at the Broad Institute for all gene set analysis. Only gene sets with a false discovery rate (FDR) *p*-value less than 0.05 and nominal *p*-values less than 0.05 were considered significantly enriched.

GSVA was performed on the 50 hallmark pathways annotated in the molecular signature database (54), which was exported using the GSEA Base package (version 1.40.1). The GSVA package (version 1.26.0) was applied with default settings to assign pathway activity estimates to individual cells.

### Single-Cell Trajectory Analysis

R package (monocle2) (24) was applied to conduct cellular trajectory analysis with the assumption that one-dimensional “time” can describe the high-dimensional expression values, the so-called pseudotime analysis of single cells. Genes for ordering cells were selected if they expressed more than 1% of the cells, their mean expression value was larger than 0.3, and dispersion empirical value was >1. Based on the “DDRTree” method, the data were reduced to two-dimensional, and then the cells were ordered along the trajectory.

## Data Availability Statement

The datasets analyzed during the current study are available in the TCGA, accession numbers TCGA-KIRC, GEO repository (GSE121636 and GSE139555).

## Author Contributions

YW, LM, and RH conceived and designed the study. YW and JL wrote the code and collected code to the github. KLX and TZ collected the data. JX and JL analyzed the data and performed the computations. ZZ, PB, and KWX wrote the manuscript. All authors contributed to the article and approved the submitted version.

## Funding

This work was supported by the China National Key Research and Development (R&D) Program (2021YFC2100100), the Hubei Provincial Department of Science and Technology Program (202110701201001), Hubei Provincial Department of Education Fund (202110701301003), Hubei University Fund (202110703000002), and the State Key Laboratory Program (SKLBEE2021012).

## Conflict of Interest

The authors declare that the research was conducted in the absence of any commercial or financial relationships that could be construed as a potential conflict of interest.

## Publisher’s Note

All claims expressed in this article are solely those of the authors and do not necessarily represent those of their affiliated organizations, or those of the publisher, the editors and the reviewers. Any product that may be evaluated in this article, or claim that may be made by its manufacturer, is not guaranteed or endorsed by the publisher.

## References

[1] Cohen HT McGovern FJ. Renal-Cell Carcinoma. N Engl J Med (2005) 353(23):2477–90. doi: 10.1056/NEJMra043172 16339096

[2] Su Y Zhang T Tang J Zhang L Fan S Zhou J. Construction of Competitive Endogenous RNA Network and Verification of 3-Key LncRNA Signature Associated With Distant Metastasis and Poor Prognosis in Patients With Clear Cell Renal Cell Carcinoma. Front Oncol (2021) 11:640150. doi: 10.3389/fonc.2021.640150 33869028 PMC8044754

[3] Filippiadis D Mauri G Marra P Charalampopoulos G Gennaro N De Cobelli F. Percutaneous Ablation Techniques for Renal Cell Carcinoma: Current Status and Future Trends. Int J Hyperthermia (2019) 36(2):21–30. doi: 10.1080/02656736.2019.1647352 31537160

[4] Krishna C DiNatale RG Kuo F Srivastava RM Vuong L Chowell D. Single-Cell Sequencing Links Multiregional Immune Landscapes and Tissue-Resident T Cells in ccRCC to Tumor Topology and Therapy Efficacy. Cancer Cell (2021) 39(5):662–77 e666. doi: 10.1016/j.ccell.2021.03.007 33861994 PMC8268947

[5] Yu EM Linville L Rosenthal M Aragon-Ching JB. A Contemporary Review of Immune Checkpoint Inhibitors in Advanced Clear Cell Renal Cell Carcinoma. Vaccines (Basel) (2021) 9(8):919 doi: 10.3390/vaccines9080919 34452045 PMC8402652

[6] Wang Y Wang Y Xu L Lu X Fu D Su J. CD4 + T Cells Promote Renal Cell Carcinoma Proliferation *via* Modulating YBX1. Exp Cell Res (2018) 363(1):95–101. doi: 10.1016/j.yexcr.2017.12.026 29289594

[7] Wu F Fan J He Y Xiong A Yu J Li Y. Single-Cell Profiling of Tumor Heterogeneity and the Microenvironment in Advanced Non-Small Cell Lung Cancer. Nat Commun (2021) 12(1):2540. doi: 10.1038/s41467-021-22801-0 33953163 PMC8100173

[8] Baslan T Hicks J. Unravelling Biology and Shifting Paradigms in Cancer With Single-Cell Sequencing. Nat Rev Cancer (2017) 17(9):557–69. doi: 10.1038/nrc.2017.58 28835719

[9] Zilionis R Engblom C Pfirschke C Savova V Zemmour D Saatcioglu HD. Single-Cell Transcriptomics of Human and Mouse Lung Cancers Reveals Conserved Myeloid Populations Across Individuals and Species. Immunity (2019) 50(5):1317–1334 e1310. doi: 10.1016/j.immuni.2019.03.009 30979687 PMC6620049

[10] Zhong R Zhang Y Chen D Cao S Han B Zhong H. Single-Cell RNA Sequencing Reveals Cellular and Molecular Immune Profile in a Pembrolizumab-Responsive PD-L1-Negative Lung Cancer Patient. Cancer Immunol Immunother (2021) 70(8):2261–74. doi: 10.1007/s00262-021-02848-0 PMC10991356 33506299

[11] Kim N Kim HK Lee K Hong Y Cho JH Choi JW. Single-Cell RNA Sequencing Demonstrates the Molecular and Cellular Reprogramming of Metastatic Lung Adenocarcinoma. Nat Commun (2020) 11(1):2285. doi: 10.1038/s41467-020-16164-1 32385277 PMC7210975

[12] Wu C Hua Q Zheng L. Generation of Myeloid Cells in Cancer: The Spleen Matters. Front Immunol (2020) 11:1126. doi: 10.3389/fimmu.2020.01126 32582203 PMC7291604

[13] Groom JR. Regulators of T-Cell Fate: Integration of Cell Migration, Differentiation and Function. Immunol Rev (2019) 289(1):101–14. doi: 10.1111/imr.12742 30977199

[14] Hu J Chen Z Bao L Zhou L Hou Y Liu L. Single-Cell Transcriptome Analysis Reveals Intratumoral Heterogeneity in ccRCC, Which Results in Different Clinical Outcomes. Mol Ther (2020) 28(7):1658–72. doi: 10.1016/j.ymthe.2020.04.023 PMC7335756 32396851

[15] Wang Q Hu J Kang W Wang J Xiang Y Fu M. Tumor Microenvironment Immune Subtypes for Classification of Novel Clear Cell Renal Cell Carcinoma Profiles With Prognostic and Therapeutic Implications. Med (Baltimore) (2021) 100(11):e24949. doi: 10.1097/MD.0000000000024949 PMC7982168 33725966

[16] Zhang Y Narayanan SP Mannan R Raskind G Wang X Vats P. Single-Cell Analyses of Renal Cell Cancers Reveal Insights Into Tumor Microenvironment, Cell of Origin, and Therapy Response. Proc Natl Acad Sci U S A (2021) 118(24):e2103240118. doi: 10.1073/pnas.2103240118 34099557 PMC8214680

[17] Borcherding N Vishwakarma A Voigt AP Bellizzi A Kaplan J Nepple K. Mapping the Immune Environment in Clear Cell Renal Carcinoma by Single-Cell Genomics. Commun Biol (2021) 4(1):122. doi: 10.1038/s42003-020-01625-6 33504936 PMC7840906

[18] Satija R Farrell JA Gennert D Schier AF Regev A. Spatial Reconstruction of Single-Cell Gene Expression Data. Nat Biotechnol (2015) 33(5):495–502. doi: 10.1038/nbt.3192 25867923 PMC4430369

[19] Aran D Looney AP Liu L Wu E Fong V Hsu A. Reference-Based Analysis of Lung Single-Cell Sequencing Reveals a Transitional Profibrotic Macrophage. Nat Immunol (2019) 20(2):163–72. doi: 10.1038/s41590-018-0276-y PMC6340744 30643263

[20] Zhang X Lan Y Xu J Quan F Zhao E Deng C. CellMarker: A Manually Curated Resource of Cell Markers in Human and Mouse. Nucleic Acids Res (2019) 47(D1):D721–8. doi: 10.1093/nar/gky900 PMC6323899 30289549

[21] Tumeh PC Harview CL Yearley JH Shintaku IP Taylor EJ Robert L. PD-1 Blockade Induces Responses by Inhibiting Adaptive Immune Resistance. Nature (2014) 515(7528):568–71. doi: 10.1038/nature13954 PMC4246418 25428505

[22] Qiu X Mao Q Tang Y Wang L Chawla R Pliner HA. Reversed Graph Embedding Resolves Complex Single-Cell Trajectories. Nat Methods (2017) 14(10):979–82. doi: 10.1038/nmeth.4402 PMC5764547 28825705

[23] Saelens W Cannoodt R Todorov H Saeys Y. A Comparison of Single-Cell Trajectory Inference Methods. Nat Biotechnol (2019) 37(5):547–54. doi: 10.1038/s41587-019-0071-9 30936559

[24] Trapnell C Cacchiarelli D Grimsby J Pokharel P Li S Morse M. The Dynamics and Regulators of Cell Fate Decisions are Revealed by Pseudotemporal Ordering of Single Cells. Nat Biotechnol (2014) 32(4):381–6. doi: 10.1038/nbt.2859 PMC4122333 24658644

[25] Deng M Bragelmann J Kryukov I Saraiva-Agostinho N Perner S. FirebrowseR: An R Client to the Broad Institute's Firehose Pipeline. Database (Oxford) (2017) 2017:baw160. doi: 10.1093/database/baw160 PMC5216271 28062517

[26] Jin S Guerrero-Juarez CF Zhang L Chang I Ramos R Kuan CH. Inference and Analysis of Cell-Cell Communication Using CellChat. Nat Commun (2021) 12(1):1088. doi: 10.1038/s41467-021-21246-9 33597522 PMC7889871

[27] Sanchez-Nino MD Sanz AB Ruiz-Andres O Poveda J Izquierdo MC Selgas R. MIF, CD74 and Other Partners in Kidney Disease: Tales of a Promiscuous Couple. Cytokine Growth Factor Rev (2013) 24(1):23–40. doi: 10.1016/j.cytogfr.2012.08.001 22959722

[28] Kawasaki H Onuki R Suyama E Taira K. Identification of Genes That Function in the TNF-Alpha-Mediated Apoptotic Pathway Using Randomized Hybrid Ribozyme Libraries. Nat Biotechnol (2002) 20(4):376–80. doi: 10.1038/nbt0402-376 11923844

[29] Jiamin C Pflieger L Sathe A Grimes S Brems M Pattison T. Identify Biomarkers Associated With Immunot oxicities Using Single-Cell RNAseq [Abstract]. In: Proceedings of the AACR Special Conference on Tumor Immunology and Immunotherapy: 2020; Boston, MA. Philadelphia. Cancer Immunol Res (2020) 8(3_supplement):A2. doi: 10.1158/2326-6074.TUMIMM19-A2

[30] Bae EA Seo H Kim IK Jeon I Kang CY. Roles of NKT Cells in Cancer Immunotherapy. Arch Pharm Res (2019) 42(7):543–8. doi: 10.1007/s12272-019-01139-8 30859410

[31] Terabe M Berzofsky JA. Tissue-Specific Roles of NKT Cells in Tumor Immunity. Front Immunol (2018) 9:1838. doi: 10.3389/fimmu.2018.01838 30158927 PMC6104122

[32] Philip M Schietinger A. CD8(+) T Cell Differentiation and Dysfunction in Cancer. Nat Rev Immunol (2021) 22(4):209–23. doi: 10.1038/s41577-021-00574-3 PMC9792152 34253904

[33] Fong L Brockstedt D Benike C Wu L Engleman EG. Dendritic Cells Injected *via* Different Routes Induce Immunity in Cancer Patients. J Immunol (2001) 166(6):4254–9:449. doi: 10.4049/jimmunol.166.6.4254 11238679

[34] Largeot A Pagano G Gonder S Moussay E Paggetti J. The B-Side of Cancer Immunity: The Underrated Tune. Cells (2019) 8(5):2228. doi: 10.3390/cells8050449 PMC6562515 31086070

[35] Dastsooz H Cereda M Donna D Oliviero S. A Comprehensive Bioinformatics Analysis of UBE2C in Cancers. Int J Mol Sci (2019) 20(9). doi: 10.3390/ijms20092228 PMC6539744 31067633

[36] Liu T Xia Q Zhang H Wang Z Yang W Gu X.CCL5-Dependent Mast Cell Infiltration Into the Tumor Microenvironment in Clear Cell Renal Cell Carcinoma Patients. Aging (Albany NY) (2020) 12(21):21809–36. doi: 10.18632/aging.103999 PMC7695370 33177244

[37] Chen JH Wu ATH Lawal B Tzeng DTW Lee JC Ho CL. Identification of Cancer Hub Gene Signatures Associated With Immune-Suppressive Tumor Microenvironment and Ovatodiolide as a Potential Cancer Immunotherapeutic Agent. Cancers (Basel) (2021) 13(15):3847. doi: 10.3390/cancers13153847 34359748 PMC8345223

[38] Seo H Gonzalez-Avalos E Zhang W Ramchandani P Yang C Lio CJ. BATF and IRF4 Cooperate to Counter Exhaustion in Tumor-Infiltrating CAR T Cells. Nat Immunol (2021) 22(8):983–95. doi: 10.1038/s41590-021-00964-8 PMC8319109 34282330

[39] Feng Y Pan L Zhang B Huang H Ma H. BATF Acts as an Oncogene in non-Small Cell Lung Cancer. Oncol Lett (2020) 19(1):205–10. doi: 10.3892/ol.2019.11075 PMC6924102 31897131

[40] Ronchetti S Ricci E Petrillo MG Cari L Migliorati G Nocentini G. Glucocorticoid-Induced Tumour Necrosis Factor Receptor-Related Protein: A Key Marker of Functional Regulatory T Cells. J Immunol Res (2015) 2015:171520. doi: 10.1155/2015/171520 25961057 PMC4413981

[41] Liu S Wang F Tan W Zhang L Dai F Wang Y.CTLA4 has a Profound Impact on the Landscape of Tumor-Infiltrating Lymphocytes With a High Prognosis Value in Clear Cell Renal Cell Carcinoma (ccRCC). Cancer Cell Int (2020) 20:519. doi: 10.1186/s12935-020-01603-2 33117084 PMC7590466

[42] Yang C Yu T Liu Z Ye X Liao X Wang X. Cystatin F as a Key Family 2 Cystatin Subunit and Prognostic Biomarker for Earlystage Pancreatic Ductal Adenocarcinoma. Oncol Rep (2019) 42(1):79–90. doi: 10.3892/or.2019.7135 31059105 PMC6549077

[43] Rogers SL Zhao Y Jiang X Eaves CJ Mager DL Rouhi A. Expression of the Leukemic Prognostic Marker CD7 Is Linked to Epigenetic Modifications in Chronic Myeloid Leukemia. Mol Cancer (2010) 9:41. doi: 10.1186/1476-4598-9-41 20175919 PMC2843654

[44] Palanichamy K Kanji S Gordon N Thirumoorthy K Jacob JR Litzenberg KT. NNMT Silencing Activates Tumor Suppressor PP2A, Inactivates Oncogenic STKs, and Inhibits Tumor Forming Ability. Clin Cancer Res (2017) 23(9):2325–34. doi: 10.1158/1078-0432.CCR-16-1323 PMC5413402 27810903

[45] Lin S Wan S Sun L Hu J Fang D Zhao R. Chemokine C-C Motif Receptor 5 and C-C Motif Ligand 5 Promote Cancer Cell Migration Under Hypoxia. Cancer Sci (2012) 103(5):904–12. doi: 10.1111/j.1349-7006.2012.02259.x PMC7659388 22380870

[46] Zhang S Zhong M Wang C Xu Y Gao WQ Zhang Y. CCL5-Deficiency Enhances Intratumoral Infiltration of CD8(+) T Cells in Colorectal Cancer. Cell Death Dis (2018) 9(7):766. doi: 10.1038/s41419-018-0796-2 29991744 PMC6039518

[47] Tang SW Yang TC Lin WC Chang WH Wang CC Lai MK. Nicotinamide N-Methyltransferase Induces Cellular Invasion Through Activating Matrix Metalloproteinase-2 Expression in Clear Cell Renal Cell Carcinoma Cells. Carcinogenesis (2011) 32(2):138–45. doi: 10.1093/carcin/bgq225 21045016

[48] Hah YS Cho HY Jo SY Park YS Heo EP Yoon TJ. Nicotinamide Nmethyltransferase Induces the Proliferation and Invasion of Squamous Cell Carcinoma Cells. Oncol Rep (2019) 42(5):1805–14. doi: 10.3892/or.2019.7315 PMC6787961 31545452

[49] Topalian SL Taube JM Anders RA Pardoll DM. Mechanism-Driven Biomarkers to Guide Immune Checkpoint Blockade in Cancer Therapy. Nat Rev Cancer (2016) 16(5):275–87. doi: 10.1038/nrc.2016.36 PMC5381938 27079802

[50] Chen Z Wang L. The Clinical Significance of UBE2C Gene in Progression of Renal Cell Carcinoma. Eur J Histochem (2021) 65(2):3196. doi: 10.4081/ejh.2021.3196 PMC8054571 33782624

[51] Wu TD Madireddi S de Almeida PE Banchereau R Chen YJ Chitre AS. Peripheral T Cell Expansion Predicts Tumour Infiltration and Clinical Response. Nature (2020) 579(7798):274–8. doi: 10.1038/s41586-020-2056-8 32103181

[52] Becht E McInnes L Healy J Dutertre CA Kwok IWH Ng LG. Dimensionality Reduction for Visualizing Single-Cell Data Using UMAP. Nat Biotechnol (2018) 37(2019):38–44. doi: 10.1038/nbt.4314 30531897

[53] Yu G Wang LG Han Y He QY. Clusterprofiler: An R Package for Comparing Biological Themes Among Gene Clusters. OMICS (2012) 16(5):284–7. doi: 10.1089/omi.2011.0118 PMC3339379 22455463

[54] Subramanian A Tamayo P Mootha VK Mukherjee S Ebert BL Gillette MA. Gene Set Enrichment Analysis: A Knowledge-Based Approach for Interpreting Genome-Wide Expression Profiles. Proc Natl Acad Sci U S A (2005) 102(43):15545–50. doi: 10.1073/pnas.0506580102 PMC1239896 16199517

