# Supplementary material for: Decoding the Immune Microenvironment of Clear Cell Renal Cell Carcinoma by Single-Cell Profiling to Aid Immunotherapy
Source: Front Immunol. 2022 Jun 24;13:791158. doi: 10.3389/fimmu.2022.791158 (PMC9263726; doi:10.3389/fimmu.2022.791158)
Supplement: Supplementary file 1 [file DataSheet_1.docx]

Supp.Fig.1-3.

Illustration of CD8+T_3 cell development trajectory inferred by SCORPIUS (Supp.Fig.1A), Slignshot (Supp.Fig.2A), TSCAN(Supp.Fig.3A), and top 50 canonical markers of each state were selected to visualize the cell development (Supp.Fig.1B-3B). Heatmap showing relative expressions of canonical markers of CD8+T_3 cells along inferred trajectories. The red and blue colors correspond to the relative gene expression level.


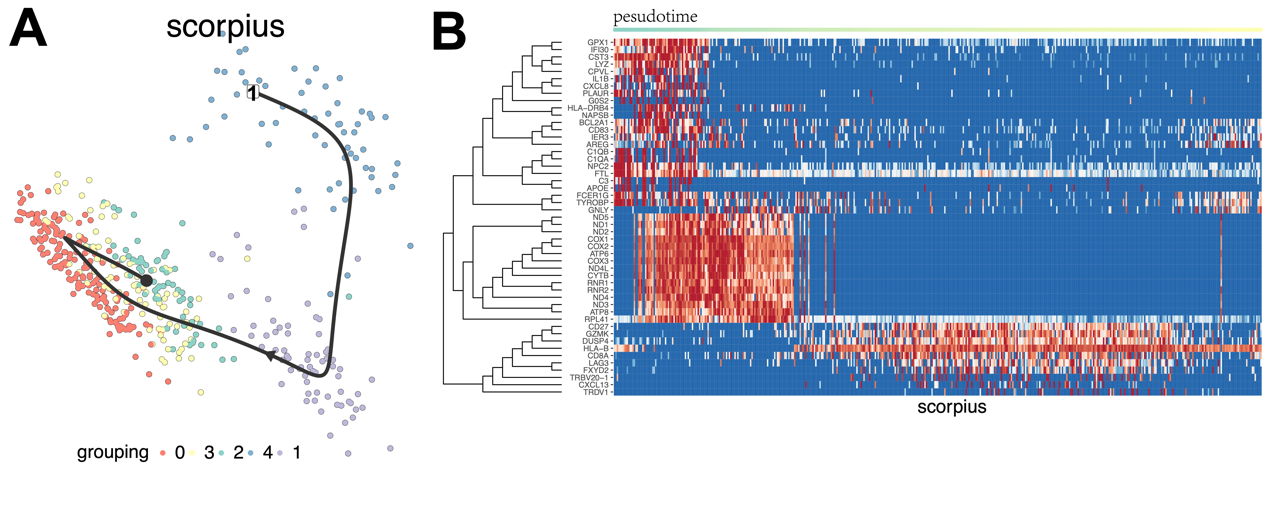


Supp.Fig.1


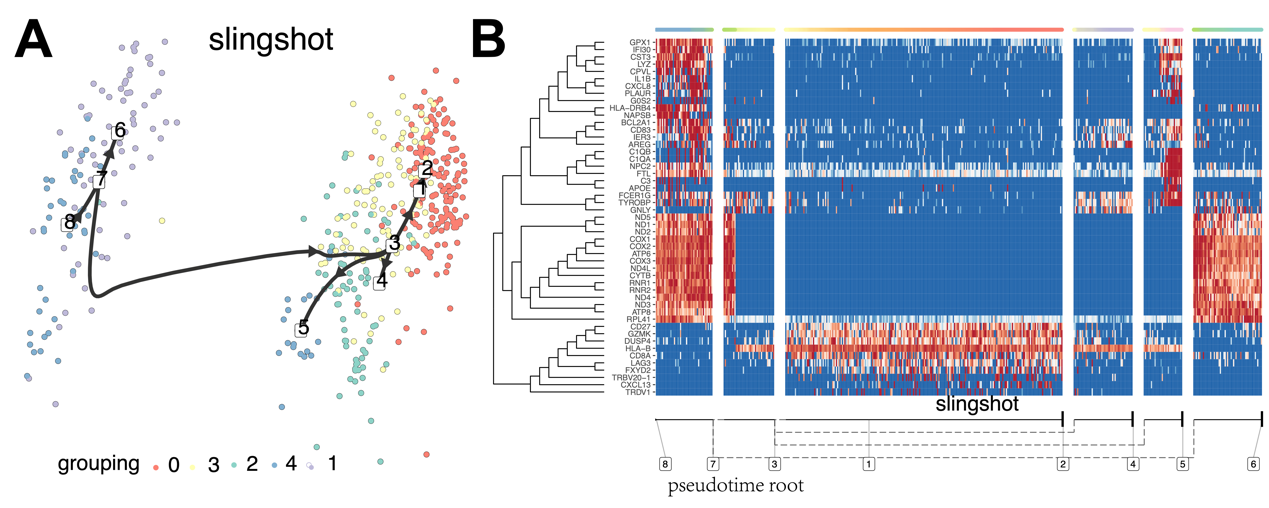


Supp.Fig.2


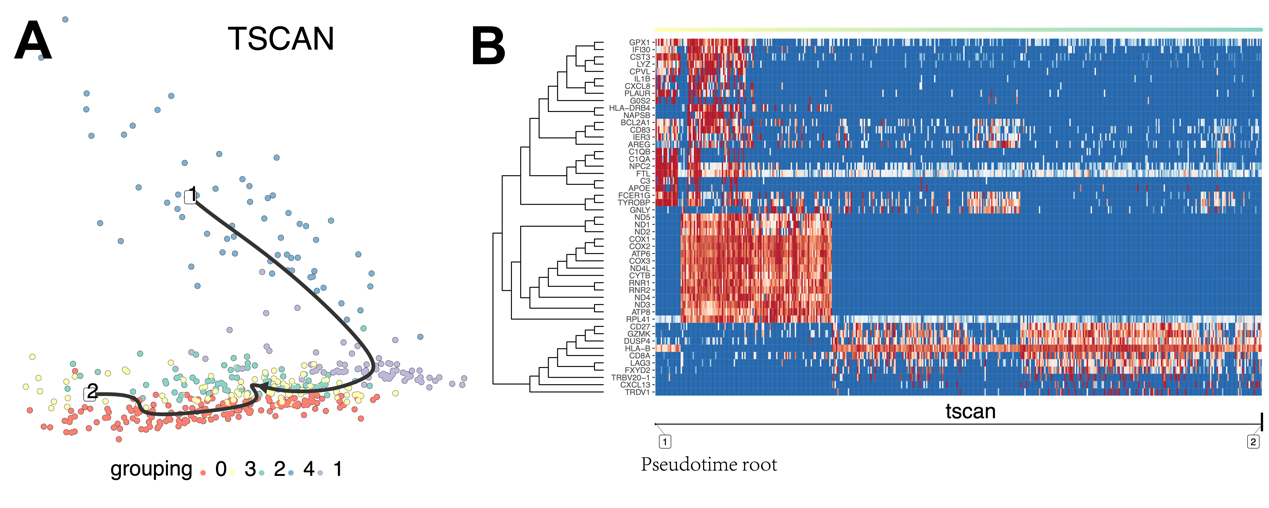


Supp.Fig.3
